# Educational Mismatch and Workers’ Fertility Intentions: Evidence from China

**DOI:** 10.3390/bs13100837

**Published:** 2023-10-13

**Authors:** Zizhe Zhang, Nan Zhao, Wanqing Liao, Hounan Chen

**Affiliations:** School of Statistics, Beijing Normal University, Beijing 100875, China; zhangzizhe@mail.bnu.edu.cn (Z.Z.); zhaonan@bnu.edu.cn (N.Z.); liaowanqing@mail.bnu.edu.cn (W.L.)

**Keywords:** educational mismatch, fertility intention, childbearing opportunity cost

## Abstract

This paper focuses on the impact of the educational mismatch on workers’ fertility intentions and explores the underlying mechanisms. Empirical research based on the China Family Panel Survey (CFPS) data shows that undereducation has a negative effect on the fertility intentions of women aged 18–35, whereas overeducation reports no effect, a finding that remains stable in the robustness test and the IV model. An explanation for this phenomenon is that undereducated female workers have a wage premium and higher expectations of career development, which implies a greater opportunity cost of fertility. The heterogeneity analysis shows that women with low socio-economic status, especially those who are less educated, from low-income households, and who are employed in the private sector, are more affected. It is therefore necessary to reduce the substitution risk in the labor market for this group and to lighten the fertility burden and pressure on women.

## 1. Introduction

Since the beginning of the 21st century, numerous nations have undergone a demographic transition characterized by “aging” and “low fertility” [1]. As the demographic crisis approaches, scholars have attempted to explore the fertility concerns afflicting individuals. An established strand of studies have found that fertility intentions are influenced by the labor market [2], economic uncertainty [3], cultural background [4], social policies [5], and many other factors. Recently, employment-related factors have gained increased prominence due to rising childcare costs and the competitive job market. Previous literature investigating the effect of educational attainment on fertility intentions has always assumed that higher educational attainment indicates better employment opportunities, thus attributing the mechanism partly to the “income effect” or “substitution effect” induced by one’s own job performance [6]. However, workers’ educational attainment does not always accurately reflect their performance in the job market. Existing studies overlook the potential change in individual fertility intentions resulting from educational mismatch.

In the labor market, the demand for human capital and the supply of workers’ qualifications are not always perfectly matched. Scholars generally regard educational attainment as a proxy for human capital and define the above phenomenon as the educational mismatch of the labor force [7]. If a worker’s educational attainment does not meet the requirements of the job, it is a case of undereducation; in the opposite case, it is a case of overeducation. The reasons for this phenomenon are complex. From a macro perspective, the fluctuations in national human capital accumulation and labor demand may force workers to accept positions that do not match their educational background [8]. At the micro level, gender, age, family background, and other attributes determine workers’ competitiveness in the job market, resulting in a mismatch [9]. Due to changes in the employment structure and education system reform, educational mismatch has become a prevalent issue in China’s labor market. According to a recent survey, only 39.7% of employees in China believe that their level of education matches the qualifications required for their current job (Annual Report on China’s Macroeconomic Situation Analysis and Forecast (2020–2021), research group of Shanghai University of Finance and Economics.).

Educational mismatch inevitably leads to several consequences, the most obvious of which is the disparity in the returns on education [7,10]. For example, if a person with a university degree takes up a simple manual job, his salary will be significantly lower than if he takes an IT job that matches his educational background. In addition, educational mismatch also affects workers’ mental health and career prospects [11]. The overeducated may feel a sense of desperation to escape career stagnation, while those who are undereducated may feel a sense of achievement and motivation to advance in their careers. These factors can intensify work–family conflict (WFC) and ultimately affect workers’ fertility intentions.

In this paper, we use data from the China Family Panel Survey (CFPS) to investigate the effect that educational mismatch has on workers’ fertility intentions. By summarizing the consequences of educational mismatch and the formation mechanism of fertility intention, we propose three potential channels. Subsequently, we conduct baseline analysis, robustness analysis, heterogeneity analysis, and mechanism analysis, and finally find a negative effect on the fertility intentions of undereducated women aged 18–35, which could be explained by childbearing opportunity cost and career development desire.

Our study broadens the established literature in three ways. First, we supplement the literature on fertility intentions. Compared with prior literature that focuses on education factors, we take individual work performance into consideration and examine the effects of overeducation and undereducation, which push the boundaries of the research. Second, we shed new light on the consequences of educational mismatch. Most of the studies find the impact of educational mismatch on manufacturers and workers. We extended the perspective from the workplace to the family and observed variances in fertility intentions caused by an educational mismatch. Third, this study is related to the literature on the opportunity cost of childbearing. By discussing the potential mechanisms, we capture not only the tangible pecuniary opportunity cost but also the intangible opportunity cost on career development and find that young undereducated women with low socio-economic status are more vulnerable.

Fertility behavior has both consumption and investment attributes. These two aspects correspond to two values: “altruism” and “intergenerational exchangeism” [12]. According to the former, children are family durable consumer goods. Individuals fulfill the role of “parent” to realize self-worth and gain social recognition [13]. The latter emphasizes intergenerational transfer payments and generalizes children as assets to provide pension security [14]. In China, traditional culture reinforces both values. On the one hand, the Chinese always pursue a strong clan, and having more children is regarded as a symbol of a happy family. On the other hand, filial piety is an important part of Chinese Confucian culture, which advocates that children give back to elderly parents. Therefore, parents are generally motivated to have children for their old age.

At the same time, fertility intentions are subject to economic budget constraints. The cost–benefit theory divides the cost of childbearing into direct and indirect cost [15]. Direct cost means that parents, constrained by their purchasing power, have to consider whether they can afford housing, food, medical care, and education for their children. Indirect cost, also known as opportunity cost, refers to the time and wages lost as a result of childbearing. In addition to the direct impact on parents’ working time, childbearing may also prevent parents from migrating in the short term or from vocational training [16], which ultimately affects work performance and results in a loss of labor income [17,18].

**Figure 1:**
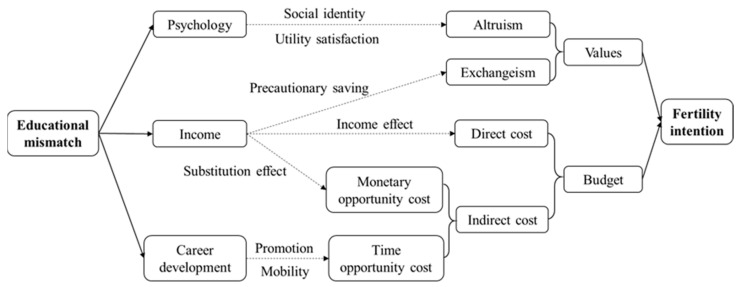
How does educational mismatch affect fertility intentions?

The degree of matching between workers’ education and their job demand reflects, to a certain extent, the quality of employment. It influences individuals’ wage level, psychological health, and career prospects, thus linking to the formation mechanism of fertility intention. In the following section, we construct a research framework that includes three potential channels through which educational mismatch may affect labor fertility intention (Figure 1).

### 1.1. Income Channel

There is a consensus that educational mismatch causes deviations in the returns on education. According to the hedonic/assignment models proposed by Sattinger [19], productivity depends on the match between the distribution of job demand and the distribution of workers’ education. Overeducation leads to worker dissatisfaction with their jobs, which increases the rate of shirking or absenteeism, and consequently causes productivity loss [20]. Evidence at the firm level demonstrates that a one-year reduction in surplus education among a company’s workers results in an 8% improvement in output [21]. Conversely, undereducation widens the potential boundaries of labor productivity and raises the upper limit of the return rate [22]. Another view is that income disparities induced by mismatches are explained by unobservable individual characteristics [23]. According to the human capital theory proposed by Becker, the marginal product of workers depends on their human capital, which consists of several factors, including education, training, work experience, and abilities [24]. Excess education can be used to compensate for deficiencies in other human capital factors, while a lack of education can be compensated for by other advantages [25,26]. In this way, productivity differences, hence income differences arise between mismatches and matches. In summary, empirical studies consistently confirm the existence of a “wage premium” associated with undereducation and a “wage penalty” associated with overeducation. More specifically, the undereducated (overeducated) individuals tend to earn more (less) than those with the same level of education who are in jobs that match their qualifications [7,27].

These income disparities may affect fertility intentions through budget constraints and value proposition: income levels determine a family’s financial well-being, affecting whether parents can afford the direct cost of childbirth and child-rearing. If children are considered normal goods, an increase in income typically results in a positive impact on fertility intentions (the wealth effect). However, income levels also impact the opportunity cost of childbearing, leading to a negative substitution effect, especially for women, who are directly involved in childbearing [28,29]. Moreover, income levels influence the potential for savings, reflecting one’s ability to prepare for future pension risks. Some scholars discover that middle-aged and elderly families with fewer children tend to have higher savings [30]. Consequently, increasing precautionary savings may offset the incentive for higher fertility as children are often seen as a form of old-age security. Accordingly, we propose Hypothesis 1.

**Hypothesis** **1.** *Education mismatch leads to income disparities, thereby influencing workers’ fertility intentions. However, the direction of this influence remains ambiguous due to the interaction of wealth effect, substitution effect, and precautionary saving.*

### 1.2. Psychological Channel

Educational mismatch also has a significant impact on the cognitive and psychological well-being of workers. Overeducated individuals usually experience feelings of exploitation, frustration, and financial insecurity. This results in lower levels of subjective well-being and an increased risk of depression compared to properly matched workers with the same number of years of schooling [31,32]. Particularly for highly educated individuals with ambitious career goals, severe overeducation can offset the positive effects of their advanced education on job satisfaction [33]. Conversely, undereducated workers tend to find self-affirmation and a sense of achievement in their work. Despite their educational disadvantage, they often report higher job satisfaction [34].

As implicit emotional capital, subjective well-being and mental health status are closely related to fertility intentions. On the one hand, according to Becker’s theory, children are perceived as a source of psychological comfort and joy in the household [35]. Parents can compensate for the stress and pressure of work with the pleasure they derive from their children. On the other hand, according to role theory, role salience represents the importance of the individual’s social roles. When an individual takes on multiple salient roles, conflicts and pressures tend to arise [36]. Therefore, work and family roles are often incompatible and the salience of one role will inhibit the performance in the other [37]. Overeducated workers may seek to build self-worth and gain social recognition through their role as ‘parents’, compensating for feelings of underachievement and loss of identity at work. In contrast, the satisfaction and well-being that undereducated workers derive from their work may reduce the desire to have children. Accordingly, we propose Hypothesis 2.

**Hypothesis** **2.** *From the psychological channel, the fertility intentions of undereducated (overeducated) workers are lower (higher) than those of the properly matched workers with the same number of years of schooling.*

### 1.3. Career Development Channel

In the long term, educational mismatch compels workers to make adjustments to their career plans. In general, undereducated individuals are considered to have long-term career development goals [33] and mostly have successful promotion experiences [38]. They expect a greater wage premium from better career development. On the other hand, overeducated individuals are more motivated to move beyond their current job status either through promotions or by actively seeking better-matched positions as soon as possible [39]. Some literature suggests that overeducation has a scarring effect on workers’ careers. Those who subjectively perceive themselves as overeducated tend to be pessimistic, show reluctance to advance in their current position, and may be more inclined to change jobs [40].

Overall, both undereducated and overeducated individuals experience career development expectations or pressures, leading to increased career mobility and uncertainty. Researchers find that job uncertainty can reduce individuals’ fertility intentions or motivate them to delay their childbearing plans [41]. Additionally, parenthood places individuals in a dual role as both ‘parents’ and ‘employees’, which imposes a time cost that hinders career advancement. Evidence suggests that women who become mothers often face career disruptions. Even after returning to the workforce after childbirth, they are less likely to attain managerial positions compared to women without children [42]. More importantly, educationally mismatched individuals need to acquire additional complementary skills, such as attending vocational training or obtaining skills certificates, to better match their positions or send a stronger signal in the job market [43]. However, childbearing prevents them from updating their skills promptly and even results in the devaluation of existing occupational skills [44]. Accordingly, we propose Hypothesis 3.

**Hypothesis** **3.** *From the career development channels, the fertility intentions of undereducated and overeducated individuals are lower than those of the properly matched workers with the same number of years of schooling.*

Previous literature highlights gender inequalities in labor supply and childbearing. As women usually take on the irreplaceable childcare responsibilities such as pregnancy and breastfeeding, their labor behaviors tend to be more fragile [45]. While a limited number of studies provide evidence of income loss for men due to paternity leave [46], traditional household divisions expose women to greater ‘work–family’ conflicts. Accordingly, we propose Hypothesis 4.

**Hypothesis** **4.** *There is a significant gender difference in the impact of educational mismatch on fertility intentions, with female workers being more susceptible than their male counterparts.*

## 2. Methods

### 2.1. Participants

The data used in this paper are from the China Household Panel Studies (CFPS). This survey has been running since 2010, tracking and collecting data at the individual, household, and community levels. The database covers 25 provinces with a target sample size of 16,000 households. We use data from the 2014 and 2018 surveys in which individuals were asked in detail about their fertility intentions.

In this study, we retain samples aged 18–50 who had full-time jobs and exclude the samples with missing values in core variables such as occupation, education, and fertility intention. Finally, 17,532 valid samples are obtained. Of these, 55% are male and the remainder are female. The mean and standard deviation of the sample age are 34.73 and 8.72, respectively. More specifically, the 18–30, 31–40, and 41–50 age groups account for 37.70%, 31.80% and 30.5%, respectively, which means that the age distribution of the sample is relatively balanced.

### 2.2. Instruments

#### 2.2.1. Educational Mismatch

Whether there is an educational mismatch is determined by two indicators: the worker’s educational attainment (*edu*) and the level of education required by the job (*redu*). When an individual’s education falls below the job requirements, they are considered undereducated (*underedu*); conversely, if an individual’s education exceeds the job requirements, they are deemed overeducated (*overedu*).

(1) $$underedu=\left\{\begin{array}{c} 1\;if\;edu<redu\\ 0\;if\;edu\geq redu \end{array}\right.$$

(2) $$overedu=\left\{\begin{array}{c} 1\;if\;redu<edu\\ 0\;if\;redu\geq edu \end{array}\right.$$

The degree of mismatch can be measured in years. Specifically, the degree of undereducation is the excess of the education required by the job (*redu*) over the education attained by the individual (*edu*), while the degree of overeducation is the excess of the education attained by the individual (*edu*) over the education required by the job (*redu*), both of which are nonnegative.

(3) $$d_{underedu}=redu-edu$$

(4) $$d_{overedu}=edu-redu$$

There are three main methods to measure educational mismatch [47]. The first is job analysis (JA), in which experts specify the *redu* of each occupation. However, there are no authoritative assessment criteria in China thus far, so it is not applicable. The second is the worker self-assessment (WA), which relies on the respondents’ subjective descriptions of whether their educational attainment matches their current occupation. Given the estimation bias caused by respondents’ vanity or subjective concealment, this is not used in this study. The third is the realized matches (RM), which calculate the average educational level of all practitioners within the occupation to estimate the *redu*, consisting of the mean method and the mode method. The former uses the mean as the criterion. If a person’s *edu* is one standard deviation higher (lower) than their *redu*, he is considered overeducated (undereducated). The latter uses the mode as the criterion and classifies one as overeducated (undereducated) if his *edu* is above (below) his *redu*. Since the realized matches effectively circumvent the drawbacks of the above two approaches, we adopt the RM estimation. In the latter part of this paper, we use the mean method to perform the baseline analysis, and the mode method is used to perform the robustness test.

In the calculation process, four points are noted:

Occupational classification. The CFPS uses the National Standard Occupational Classification and Code of the People’s Republic of China (GB/T6565-2009) [48], which divides all occupations into 8 major categories, 65 medium categories, and 410 subcategories. In this paper, we use the medium categories as the basis for classification. To avoid the statistical bias caused by the small sample size, we merge the medium categories with similar *redu* that contain less than 30 samples under the same major category. If there is no similar category, the small sample will be excluded.

Generation differences. In the late 1990s, China began to implement the college enrollment expansion plan. Since then, higher education has become popularized, and the scale of college students has expanded rapidly. Correspondingly, the labor market also imposes strict requirements on the education of employees. It is unreasonable to directly take the average educational level of all-age employees as the criterion required for occupation. In this regard, we divided the sample into two generations born around the 1980s and measured the *redu* of the two generations.

Regional differences. There are significant differences in the educational attainment of workers from different regions. According to data from the Seventh National Population Census in 2020, the average number of years of schooling for people aged 15 and over is 9.91 years nationwide. However, there are significant disparities, with an average of 12.64 years in Beijing and 8.75 years in Guizhou. There is also a significant regional economic divide between China’s eastern, central, and western regions, which leads to different demands on human capital. For example, when looking at the education level of purchasing and sales personnel, the averages in the eastern, central, and western regions are 10.42, 10.10 and 9.57 years, respectively. We therefore divide the sample into three segments based on their geographical location.

Heterogeneity of education within occupations. Considering the technological refinement and organizational structure settings, the same occupation requires workers with different qualifications to work together, which is ignored by the RM approach [49]. Accordingly, we introduce the Index of Qualitative Variation (*IQV*) [50] into the prediction equation for fertility intention to control the educational heterogeneity within occupations. The calculation formula is as follows:

(5) $$IQV=\frac{K(N^{2}-\sum f^{2})}{N^{2}(K-1)}$$

where *K* is the number of education categories, *N* is the number of individuals within the occupation, and *f* is the number of individuals within each education category within the occupation.

Based on the above principles, we divide all the workers into several groups, calculate the average education level within each group, and determine whether each sample falls into the categories of undereducated, overeducated, or educationally matched (the *redu* estimates within each group are shown in Table A1). The calculations reveal that the undereducated group comprises 9.54% of the sample, whereas the overeducated group accounts for 17.01%.

#### 2.2.2. Fertility Intention

Research on fertility decisions tends to focus on fertility intentions and actual fertility behavior. However, since an individual’s career trajectory may change after childbirth, it is difficult to accurately determine the educational mismatch of respondents before childbirth. In this paper we focus on the fertility intentions of workers. In previous studies, the intended number of children is usually used to measure fertility intentions [51]. The CFPS survey asked, “How many children do you expect to have?” The survey results show that the average intended number of children is 1.82, with 1.16%, 23.15% and 69.55% of respondents answering 0, 1 and 2, respectively.

### 2.3. Data Collection Procedure

CFPS is conducted by the Institute of Social Science Survey (ISSS) at Peking University. In 2010, the CFPS officially launched baseline surveys in 25 provinces, municipalities, and autonomous regions across the country, eventually interviewing 14,960 households and 42,590 individuals. All family members identified in the baseline survey, along with their future biological or adopted children, are considered CFPS genetic members and serve as permanent subjects for CFPS surveys conducted every two years.

CFPS uses computer-assisted personal interviewing (CAPI), this the first time this approach has been used in a large-scale longitudinal project in China. During each round of the survey, the CFPS project team recruits a significant number of local interviewers in the sample areas, brings these interviewers to Peking University for standardized training, and conducts a nationwide household interview survey using the CAPI mode. Throughout the survey, interviewers are required to send data back to ISSS on a daily basis after completing the household interviews in the field.

### 2.4. Data Analysis

This study uses Stata version 16.0 for data analysis. We sequentially perform data description, baseline regression analysis, robustness test, mechanism analysis, and heterogeneity analysis to explore how educational mismatch affects workers’ fertility intentions.

## 3. Results

### 3.1. Model

The empirical model is as follows:

(6) $${fert}_{ijmt}=\alpha +\beta {d_{mismatch}}_{ijmt}+{\gamma edu}_{ijmt}+\rho X+\mu_{j}+\theta_{m}+\lambda_{t}+\varepsilon_{ijmt}$$

where the dependent variable ${fert}_{ijmt}$ denotes the fertility intention of worker i in industry m in county *j* in year *t*. The independent variable ${d_{mismatch}}_{ijmt}$ is the degree of educational mismatch, which is manifested in the form of ${d_{underedu}}_{ijmt}$ and ${d_{overedu}}_{ijmt}$, respectively. ${edu}_{ijmt}$ indicates educational attainment. X are control variables including gender, age, hukou, residence, health status, marital status, employment break, family size, the proportion of the household who are elderly, and household income, the *IQV* index. The regression also includes fixed effects for industry, county, and year. $\varepsilon_{ijmt}$ is the disturbance term.

When examining the effects of undereducation (overeducation), we exclude overeducated (undereducated) workers and include only those who are properly matched as the control group. As a result, the estimation of *β*, which we are interested in, captures the differences in fertility intentions among undereducated (overeducated) workers compared to those who are properly matched with the same number of years of schooling. Given that educational matches seldom change in the short term, the variation over time may not be significant enough to be captured by the fixed-effect model [52]. We use ordinary least squares estimation (OLS). The definitions and descriptive statistics of the variables involved are shown in Table 1. The correlation matrix is shown in Table A2.

### 3.2. Baseline Regression

The benchmark results are shown in Table 2. Given that individuals have gender characteristics and age-appropriate fertility stages, we divide the samples according to gender and age. Panel A and Panel B are the estimations of undereducation and overeducation, respectively. Column (1) of Table 2 is the estimation of the full sample, and columns (2)–(5) of Table 2 are the estimations of the female and male samples aged 18–35 and 36–50, respectively.

As we can see from the estimations of the whole sample, educational mismatch does not affect workers’ fertility intentions. However, the results show significant heterogeneity after sample grouping. One is gender heterogeneity, and the other is age heterogeneity. Specifically, educational attainment has a negative effect on the fertility intentions of female workers aged 18–35. Moreover, if they are undereducated, fertility intentions will be further reduced. At the 5% significance level, the fertility intentions of women aged 18–35 decrease by 0.53% for each year of undereducation (the average fertility intention of women aged 18–35 is 1.751, so the impact of undereducation is 0.0092/1.751 × 100% = 0.53%), while overeducation reports no significant effect on fertility intentions.

### 3.3. Robustness Test

The benchmark result may have serious endogeneity. First, measurement error, using different measures obtains different estimates of educational mismatch. Second, omitted variable bias, fertility intentions imply individual feelings and values that are difficult to quantify or introduce into the model. Third, due to self-selection bias, some individuals take their fertility intentions into consideration, thus having a preference for specific occupations and choosing jobs that do not fit their educational background. We adopt a series of robustness tests to ensure that the findings are robust and reliable.

#### 3.3.1. Discussion about Age Differences

The baseline regression restricts the sample to only two groups above and below the age of 35, which cannot identify the effect of educational mismatch across age groups in detail. Taking 5 years as a stage, we divide the sample age into six generations and set dummy variables for each generation group (${age\_c}_{ijmt}$). The following model examines the interaction of educational mismatch and generation on fertility intention, with 46–50 years old as the control group.

(7) $${fert}_{ijmt}=\alpha +\sum_{c=1}^{6}\beta_{c}{d_{mismatch}}_{ijmt}\times {age\_c}_{ijmt}+{\gamma edu}_{ijmt}+\rho X+\mu_{j}+\theta_{m}+\lambda_{t}+\varepsilon_{ijmt}$$

**Figure 2:**
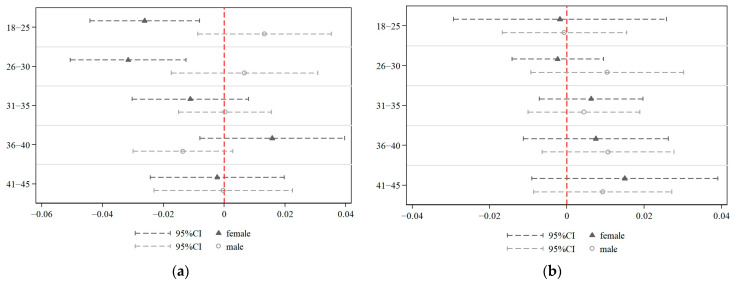
The intergenerational effect of educational mismatch: (**a**) depicts the effect of undereducation; (**b**) depicts the effect of overeducation.

Figure 2 plots the $\beta_{c}$ estimates and 95% confidence intervals for undereducation and overeducation, where the horizontal axis represents the point estimate, and the vertical axis represents the age group. Figure 2a shows that the negative effect of undereducation on female fertility intentions is mainly found among the 18–30 age group. No significant effect of overeducation on female fertility intention is observed (Figure 2b). This finding is consistent with the results of the benchmark.

#### 3.3.2. Discussion about Measurement Error

- Dummy variables. We transform the explanatory variable from the continuous variable of educational mismatch years to the 0–1 dummy variable of whether it is undereducated/overeducated. After substituting dummy variables, the results in columns (1)–(2) of Table 3 are consistent with those for continuous variables.
- Mode method. Furthermore, we replace the educational mismatch measured by the mean method with the measurement of the mode method for verification. The results in columns (3)–(4) of Table 3 confirm that undereducation has a negative impact on the fertility intentions of women.

#### 3.3.3. Discussion about the Omitted Variable Bias

- Traditional gender norms. Gender norms can significantly influence both women’s labor participation and childbearing behaviors. On one hand, traditional gender norms often dictate that women should assume greater responsibilities for household work rather than engaging in productive labor. On the other hand, these norms emphasize the importance of family continuity, placing a significant burden on women for reproduction. The decline in fertility intentions among undereducated women can be attributed to the increasingly egalitarian gender norms. These changing norms encourage women to pursue higher positions within the workplace, potentially leading them to prioritize their careers over childbearing. The CFPS survey asked, “How important are family and reproduction, scored from 1–5?” We introduce this answer as the proxy of the views on traditional gender norms.
- Fertility status. Current fertility status may influence both employment choices and future fertility intentions. Previous studies have shown that when women have children to care for, they tend to be overeducated in order to balance their family and career [53]. In addition, the experience of parenthood may influence fertility intentions [54]. Both qualitative and quantitative research has shown that the physical and socio-psychological experiences of childbearing and childrearing, particularly the subjective well-being associated with the first birth, play an important role in predicting future fertility plans [55,56]. We therefore introduce fertility status into the model.
- Migration status. Migration status can also be an omitted variable in the benchmark regression. Workers often migrate in search of better job prospects, especially in the face of the rapid expansion of the market economy and accelerated urbanization. There are more opportunities for migrant workers to advance their careers in the place of relocation. Nevertheless, ensuring equitable access to public resources and services for the migrant population remains a challenge, which may contribute to lower fertility intentions among this group. To eliminate the interference brought by migration, we further control the relevant variables in the benchmark model.
- In Table 4, columns (1) and (5), (2) and (6), and (3) and (7) present the results considering the traditional gender norm, fertility status, and migration status, respectively. Columns (4) and (8) of Table 4 are the results considering the above omitted variables at the same time. As shown in Table 4, the traditional gender norm and fertility experience can indeed significantly improve workers’ fertility intentions. However, after controlling for the above variables, the effect of undereducation on women aged 18–35 is still significant.

#### 3.3.4. An IV Model

The instrumental variable (IV) is an alternative approach for endogeneity. In this paper, we select the proportion of province-intergenerational technical personnel and the operational stock of the intra-industry robots (data source: International Federation of Robotics (IFR)) as instrumental variables. The reasons are as follows.

First, the proportion of province-intergenerational professionals reflects the demand for highly skilled workers among different regions, and this demand is closely linked to the educational distribution of workers. In general, regions with a higher proportion of technical professionals tend to attract a more technology-intensive workforce, which is more likely to be undereducated rather than overeducated.

Second, the wave of artificial intelligence and robotics has fundamentally reshaped the labor market. According to the task-based model, scholars contend that the implementation of automation technology affects labor demand by either replacing human resources or generating new job opportunities [57]. From an industrial development perspective, it typically requires a certain amount of time to mass-produce new occupations with the integration of robotics. In the short term, the influx of robots can disrupt the job market, resulting in job displacement and narrowing the labor demand gap in related industries. This situation leads to an increase in overeducation rather than undereducation.

Third, educational mismatch depends on demand–supply interactions. From the perspective of the supply side, individual employment decisions are strongly influenced by self-induced factors, which makes it difficult to remove self-selection bias. It is necessary to select instrumental variables from the more exogenous demand side. The two instrumental variables selected in this paper reflect the demand for human capital from two macro levels, namely, the employment structure across regions and the technological trend within the industry. They determine the probability of being undereducated or overeducated in the labor market, but are generally unaffected by individuals’ fertility intentions.

In summary, the above instrumental variables satisfy the conditions of relevance and exogeneity. Table 5 reports the results of two-stage least square estimation (2SLS), where Panel A and Panel B are the estimations of undereducation and overeducation, respectively. The auxiliary test of instrumental variables shows that the F-value test rejects the weak correlation hypothesis. The *p*-values of Hansen J statistics in the overidentification test are all above 0.05, indicating that the instrumental variables are exogenous. The first-stage regression results show that a higher proportion of province-intergenerational technical personnel is related to more undereducation and less overeducation. Robotics mainly substitutes for female workers over 35 years old. More precisely, it reduces the probability of undereducation and enhances the possibility of overeducation. The second-stage estimations show that the fertility intentions of 18–35 years old undereducated women are significantly lower than those of the properly matched workers with the same number of years of schooling, which confirms the benchmark.

#### 3.3.5. Propensity Score Matching

As mentioned above, sample self-selection challenges the research findings. Individuals with stronger fertility intentions and a higher commitment to family life tend to gravitate towards less demanding jobs. Conversely, those with lower fertility aspirations and higher career aspirations are more likely to seek more lucrative employment opportunities. In this case, it is difficult to discern the effect of educational mismatches on fertility intentions.

In this paper, we use propensity score matching (PSM) to construct a counterfactual test. The principle of PSM is to match the treatment group with the control group that has similar characteristics to guarantee comparability. The difference between the two groups is the average treatment effect. In the analysis, the educationally mismatched workers are taken as the treatment group, while the properly matched workers are taken as the control group. We adopt k-nearest neighbor matching, radius matching, local linear regression matching, and kernel matching to match the treatment group with the control group and calculate the average treatment effects. On the basis of previous findings, we mainly studied women aged 18–35. As can be seen in Table 6, the results obtained by the PSM conclude that for women aged 18–35, undereducation has a significantly negative effect on their fertility intentions.

### 3.4. Mechanism Analysis

Why does undereducation have a negative impact on young women’s fertility intentions? This section verifies the three potential channels listed in the theoretical analysis.

The income channel hypothesis suggests that there is a “wage premium” for undereducation, which increases the opportunity cost of childbearing or increases precautionary saving for old age. Column (1) of Table 7 shows that undereducation has a significant effect on workers’ wages. However, does this wage premium translate into higher household savings? We conducted additional tests, but did not find a significant effect on household savings. Given that our focus is on young women aged 18–35, a demographic group that is less likely to have developed expectations about retirement situations in the distant future, the path of precautionary savings does not seem to hold.

The psychological channel hypothesis suggests that undereducated workers generally have a high sense of achievement and job satisfaction, which strengthens their perception of their role at work and weakens their desire to have children. As shown in column (3) of Table 7, the result reports no significant effect on women’s job satisfaction. To confirm this finding, we introduced subjective well-being as a proxy for workers’ intrinsic emotions, as shown in column (4) of Table 7. The result also reports that undereducation has no significant effect on employees’ well-being. Therefore, the psychological channel hypothesis cannot explain the effect on women’s fertility intentions.

The career development hypothesis posits that undereducation influences women’s fertility intentions by reshaping their career plans. To examine this, we introduced a dummy variable indicating workers’ promotion intentions. This variable takes the value of one if the worker aims to attain a technical title or seek promotion to an administrative role; otherwise, it is set to zero. Columns (5)–(7) of Table 7 reveal that undereducated women exhibit a stronger desire for career advancement, particularly in their pursuit of technical titles.

### 3.5. Heterogeneity Analysis

This paper finds that women aged 18–35 are affected by educational mismatch. Within this group, what kind of women are more likely to be affected? In this section, we discuss the heterogeneity in the results depending on the characteristics of the female worker and her family.

First, we divided the samples into two groups, low-educated and high-educated, based on whether the workers had completed high school education or more. As confirmed in columns (1) and (2) of Table 8, this effect is particularly pronounced for low-educated women, while it is not statistically significant for high-educated women.

Then, based on household income quantiles, we divided the sample into three groups: low-income, middle-income, and high-income. As depicted in Columns (3)–(5) of Table 8, undereducation only exerts an impact on women from low-income households.

Furthermore, as can be seen in columns (6) and (7) of Table 8, the negative effect of undereducation on the fertility intentions of female workers mainly affects those who are employees, while the self-employed group shows an opposite trend. To explore the details, we divide employees into subgroups according to whether they work in the public or private sector. The results in columns (8) and (9) of Table 8 show that undereducated women employed in private enterprises, foreign-funded enterprises, and other private sectors with a higher risk of termination due to childbearing, have significantly lower fertility intentions. Conversely, those employed in government agencies, state-owned enterprises, or public institutions with stable employment conditions remain unaffected. This supports the previous hypothesis to some extent.

## 4. Discussion

### 4.1. Effect of Educational Mismatch on Workers’ Fertility Intentions

Using CFPS data, we first examine the impact of educational mismatch on workers’ fertility intentions. Our research confirms that undereducation has a negative impact on the fertility intentions of female workers aged 18–35. Specifically, fertility intentions decrease by an average of 0.53% per year of undereducation, while overeducation reports no significant effect. These findings remain robust when accounting for factors such as measurement error, omitted variable bias, self-selection bias, and the use of IV and PSM methods. Previous studies have generally found that higher education levels are associated with lower fertility intentions among women [58,59]. Our study extends this understanding by suggesting that women whose job requirements exceed their education level have even lower fertility intentions.

Why are women aged 18–35 years old influenced by the educational mismatch? According to the 2015 National Population Sample Survey, the fertility rate among women of childbearing age in China is 30.93%, with an average fertility rate of 53.92% for women aged 18–35. This demographic group is at a pivotal point in their lives, marked by both childbearing and career development. In contrast to men, women often experience double pressure, both from the labor market and family. They bear the responsibility for material production, such as paid employment, while also shouldering the role of social reproduction, which includes unpaid domestic work. Educational mismatch in the labor market may further intensify the conflict between family and work.

### 4.2. Mechanism for the Effect of Educational Mismatch on Workers’ Fertility Intentions

We then explore the potential channels for the negative effect of undereducation on the fertility intentions of women aged 18–35.

On the one hand, it is the tangible opportunity cost (in the form of income penalties) that contributes to the decline in fertility intentions among undereducated women. We find that compared with individuals whose employment is properly matched with the same number of years of schooling, undereducated individuals have a wage premium. Numerous studies find that women may experience career interruptions and wage decline after childbirth, which is also known as a maternal penalty [45]. Moreover, these penalties tend to be more pronounced for women with lower levels of education [60]. Among female workers aged 18–35, the average number of years of schooling for the undereducated, the properly matched and the overeducated are 7.56, 11.92 and 15.31, respectively, indicating that undereducation is predominantly observed among those with lower levels of education. Consequently, educational mismatch reduces the fertility intentions of female workers by increasing the opportunity cost of childbearing.

On the other hand, the intangible opportunity cost (in the form of limited career advancement) plays a key role. Compared with individuals whose employment is properly matched with the same number of years of schooling, undereducated individuals have higher expectations of career development. Previous research indicates that childbirth often disrupts the accumulation of work experience and professional skills, diminishing women’s competitiveness for career progression [44,61]. In essence, undereducated female workers tend to harbor higher expectations for career advancement. However, due to the potential disengagement from the job market associated with childbirth, they may subsequently reduce their desire to have children.

### 4.3. Heterogeneous Effects on Different Groups

The above underlying mechanism is confirmed through heterogeneity analysis. We find that women with low socio-economic status are more likely to reduce their fertility intentions due to undereducation.

In terms of educational attainment, low-educated women are more affected. Since low-educated female workers face a higher risk of substitution and face more severe challenges when returning to work after childbirth, they are more reluctant to give up the good employment opportunities that undereducation brings, thereby reducing their fertility intentions.

In terms of family income, women from low-income families are more affected. This confirms the hypothesis that undereducated women tend to have weaker fertility intentions due to the opportunity cost of childbearing. Given that low-income families often have limited financial resources, they may struggle more to accommodate the loss of wage premium resulting from an undereducated family member’s temporary departure from the workforce.

In terms of employment type, women who are employed in the private sector are more affected. This finding may be attributed to the fact that, compared to the self-employed, employees face the risk of being dismissed due to childbearing. Furthermore, unlike the family-friendly public sector, the private sector tends to be less accommodating to female employees who are pregnant or have children [62,63].

### 4.4. Limitations

This study has several limitations. First, our model fails to include work experience due to data constraints. Previous research has indicated that undereducation and work experience are often mutually substitutable, which may explain wage premiums. In the future, when the data is available, the inclusion of work experience as a control variable could potentially increase the strength of the conclusions. Second, our analysis primarily focuses on discussing the impact of educational mismatch on workers’ fertility intentions. It might be more meaningful to examine the effect of educational mismatch on actual fertility behavior, including the number of children and the timing of childbirth. However, we could only obtain information about workers’ current jobs and lack historical work trajectories before childbirth. Consequently, conducting such research is challenging, as many people, especially women, change jobs after giving birth.

## 5. Conclusions

Our study finds a negative effect on the fertility intentions of undereducated women aged 18–35, which could be explained by childbearing opportunity cost and career development desire. Specifically, women with low socio-economic status are more affected. For these groups, the undereducation premium holds even greater significance. At the same time, they face a higher likelihood of labor market replacement after childbirth.

Our findings have the following implications. On the one hand, online education, distance education, and other flexible ways can be adopted to provide learning opportunities for the undereducated group to enhance the degree of matching between their qualifications and job requirements, thus alleviating their substitution risks in the labor market. On the other hand, the protection of women’s rights and interests in the labor market, such as the establishment of diversified maternity allowance systems and the provision of post-natal employment counselling, should be accelerated to remove the reproductive burden and opportunity cost faced by women.

## Figures and Tables

**Table 1:** Descriptive Statistics.

Variable	Mean	Std	Min	Max
Intended number of children	1.82	0.64	0	12
Degree of undereducation	0.54	1.77	0	14.11
Degree of overeducation	0.81	1.91	0	11.27
Years of educational attainment	10.55	3.85	0	22
Gender (male = 1, female = 0)	0.55	0.50	0	1
Age	34.73	8.72	18	50
Hukou (rural hukou = 1, urban hukou = 0)	0.63	0.48	0	1
Residence (urban = 1, rural = 0)	0.64	0.48	0	1
Health status (score of 1–5)	2.66	1.06	1	5
Marital status (unmarried = 1; married = 2; divorced = 3)	1.86	0.43	1	3
Employment break (yes = 1, no = 0)	0.29	0.46	0	1
Family size	4.19	1.83	1	10
The proportion of household members aged 65	0.07	0.14	0	0.86
Total household income (log)	10.96	0.87	0	15.27
IQV index	0.86	0.03	0.75	0.96
Views on traditional gender norms (score of 1–5)	3.39	1.45	1	5
Fertility status (have children = 1, childless = 0)	0.75	0.43	0	1
Migration (migration across city = 1, else = 0)	0.11	0.31	0	1
Wage (log)	10.26	0.89	0	13.64
Household savings (log)	7.04	5.03	0	15.43
Job satisfaction (score of 1–5)	3.52	0.83	1	5
Subjective well-being (score of 0–10)	7.60	2	0	10
Willing to promotion (yes = 1, no = 0)	0.58	0.49	0	1
Employment type (employed = 1, self-employed = 0)	0.80	0.40	0	1

**Table 2:** Benchmark results.

Variables		18–35		36–50	
	All	Female	Male	Female	Male
	(1)	(2)	(3)	(4)	(5)
**Panel A**					
$d_{underedu}$	0.0008	−0.0092 **	0.0088	0.0089	−0.0082
	(0.0032)	(0.0040)	(0.0088)	(0.0060)	(0.0105)
edu	−0.0043	−0.0090 **	−0.0102	−0.0011	−0.0010
	(0.0027)	(0.0041)	(0.0069)	(0.0049)	(0.0067)
Controls	Yes	Yes	Yes	Yes	Yes
Industry FE	Yes	Yes	Yes	Yes	Yes
County FE	Yes	Yes	Yes	Yes	Yes
Year FE	Yes	Yes	Yes	Yes	Yes
Observations	14,535	3699	3852	2939	3626
R-squared	0.1997	0.2141	0.2133	0.2944	0.2839
**Panel B**					
$d_{overedu}$	0.0030	0.0007	0.0052	−0.0015	0.0043
	(0.0027)	(0.0045)	(0.0052)	(0.0087)	(0.0051)
edu	−0.0020	−0.0114 ***	−0.0028	0.0019	0.0019
	(0.0023)	(0.0027)	(0.0043)	(0.0047)	(0.0047)
Controls	Yes	Yes	Yes	Yes	Yes
Industry FE	Yes	Yes	Yes	Yes	Yes
County FE	Yes	Yes	Yes	Yes	Yes
Year FE	Yes	Yes	Yes	Yes	Yes
Observations	15,830	3854	4437	2983	4095
R-squared	0.1879	0.2060	0.1912	0.2761	0.2675

Notes: Robust standard errors, reported in parentheses, are clustered by industry and county. ***, and ** denote significance at the 1%, and 5% levels, respectively. The detailed version, which includes the estimated results of the control variables, is presented in Table A3.

**Table 3:** Results of replacing the explanatory variable.

Variables	Dummy Variables		Mode Method	
	Female	Male	Female	Male
	(1)	(2)	(3)	(4)
**Panel A**				
underedu	−0.0318 *	0.0220		
	(0.0182)	(0.0446)		
$d_{underedu}$			−0.0152 **	−0.0018
			(0.0054)	(0.0099)
edu	−0.0079 *	−0.0124 *	−0.0154 ***	−0.0194 **
	(0.0043)	(0.0061)	(0.0037)	(0.0090)
Controls	Yes	Yes	Yes	Yes
Industry FE	Yes	Yes	Yes	Yes
County FE	Yes	Yes	Yes	Yes
Year FE	Yes	Yes	Yes	Yes
Observations	3699	3852	2833	2824
R-squared	0.2137	0.2131	0.2308	0.2367
**Panel B**				
overedu	0.0017	0.0209		
	(0.0186)	(0.0211)		
$d_{overedu}$			0.0026	0.0053
			(0.0035)	(0.0036)
edu	−0.0113 ***	−0.0022	−0.0105 ***	−0.0043
	(0.0030)	(0.0041)	(0.0034)	(0.0044)
Controls	Yes	Yes	Yes	Yes
Industry FE	Yes	Yes	Yes	Yes
County FE	Yes	Yes	Yes	Yes
Year FE	Yes	Yes	Yes	Yes
Observations	3854	4437	3260	3881
R-squared	0.2060	0.1911	0.2144	0.1953

Notes: The above table lists the estimates of the sample aged 18–35. Robust standard errors, reported in parentheses, are clustered by industry and county. ***, **, and * denote significance at the 1%, 5%, and 10% levels, respectively. The detailed version, which includes the estimated results of the control variables, is presented in Table A4.

**Table 4:** Discussion about the omitted variable bias.

Variables	Female				Male			
	(1)	(2)	(3)	(4)	(5)	(6)	(7)	(8)
**Panel A**								
$d_{underedu}$	−0.0087 *	−0.0090 **	−0.0088 **	−0.0081 *	0.0094	0.0089	0.0115	0.0122
	(0.0043)	(0.0039)	(0.0038)	(0.0039)	(0.0088)	(0.0089)	(0.0104)	(0.0104)
edu	−0.0063	−0.0081 *	−0.0092 **	−0.0063	−0.0074	−0.0097	−0.0113	−0.0083
	(0.0040)	(0.0042)	(0.0039)	(0.0040)	(0.0067)	(0.0070)	(0.0085)	(0.0086)
gen_view	0.0370 ***			0.0281 ***	0.0512 ***			0.0463 ***
	(0.0061)			(0.0065)	(0.0083)			(0.0098)
fer_status		0.0732 **		0.0744 **		0.0585		0.0474
		(0.0300)		(0.0268)		(0.0338)		(0.0292)
mig_status			0.0242	0.0259			−0.0244	−0.0222
			(0.0371)	(0.0364)			(0.0466)	(0.0446)
Controls	Yes	Yes	Yes	Yes	Yes	Yes	Yes	Yes
Industry FE	Yes	Yes	Yes	Yes	Yes	Yes	Yes	Yes
County FE	Yes	Yes	Yes	Yes	Yes	Yes	Yes	Yes
Year FE	Yes	Yes	Yes	Yes	Yes	Yes	Yes	Yes
Observations	3698	3699	3320	3319	3852	3852	3331	3331
R-squared	0.2207	0.2155	0.2172	0.2226	0.2207	0.2138	0.2298	0.2366
**Panel B**								
$d_{overedu}$	0.0008	0.0010	−0.0010	−0.0004	0.0048	0.0052	0.0073	0.0066
	(0.0045)	(0.0045)	(0.0050)	(0.0049)	(0.0053)	(0.0051)	(0.0055)	(0.0056)
edu	−0.0081 ***	−0.0106 ***	−0.0119 ***	−0.0081 ***	−0.0004	−0.0023	−0.0049	−0.0017
	(0.0024)	(0.0027)	(0.0028)	(0.0027)	(0.0044)	(0.0043)	(0.0057)	(0.0057)
gen_view	0.0451 ***			0.0369 ***	0.0495 ***			0.0454 ***
	(0.0076)			(0.0083)	(0.0090)			(0.0101)
fer_status		0.0544 *		0.0560 *		0.0539 *		0.0622 *
		(0.0308)		(0.0298)		(0.0289)		(0.0323)
mig_status			0.0238	0.0201			−0.0154	−0.0165
			(0.0329)	(0.0326)			(0.0399)	(0.0387)
Controls	Yes	Yes	Yes	Yes	Yes	Yes	Yes	Yes
Industry FE	Yes	Yes	Yes	Yes	Yes	Yes	Yes	Yes
County FE	Yes	Yes	Yes	Yes	Yes	Yes	Yes	Yes
Year FE	Yes	Yes	Yes	Yes	Yes	Yes	Yes	Yes
Observations	3854	3854	3451	3451	4437	4437	3825	3825
R-squared	0.2152	0.2069	0.2099	0.2171	0.1983	0.1916	0.1988	0.2059

Notes: The above table lists the estimates of the sample aged 18–35. Robust standard errors, reported in parentheses, are clustered by industry and county. ***, **, and * denote significance at the 1%, 5%, and 10% levels, respectively. The detailed version, which includes the estimated results of the control variables, is presented in Table A5.

**Table 5:** Results of 2SLS.

Variables		18–35		36–50	
	All	Female	Male	Female	Male
	(1)	(2)	(3)	(4)	(5)
**Panel A**					
$d_{underedu}$	0.0375	−0.0651 ***	0.0376	0.0026	−0.0227
	(0.0246)	(0.0146)	(0.0771)	(0.0596)	(0.0476)
edu	0.0121	−0.0336 ***	0.0022	−0.0043	−0.0082
	(0.0116)	(0.0084)	(0.0312)	(0.0290)	(0.0249)
First-stage					
tech_p	7.4118 ***	8.2680 ***	7.5607 ***	8.6787 ***	7.6972 ***
	(0.9073)	(1.4122)	(1.5857)	(1.2210)	(0.9190)
rob	−0.0025	−0.0058	−0.0012	−0.0079 **	−0.0010
	0.0015	(0.0049)	(0.0020)	(0.0040)	(0.0010)
Controls	Yes	Yes	Yes	Yes	Yes
Industry FE	Yes	Yes	Yes	Yes	Yes
County FE	Yes	Yes	Yes	Yes	Yes
Year FE	Yes	Yes	Yes	Yes	Yes
F statistic	34.06	17.68	14.18	26.98	46.38
Hanse J *p* value	0.252	0.190	0.506	0.181	0.493
Observations	14488	3685	3841	2927	3615
**Panel B**					
$d_{overedu}$	−0.0130	0.0331	−0.0010	0.0342	0.0212
	(0.0172)	(0.0196)	(0.0509)	(0.0583)	(0.0299)
edu	0.0048	−0.0250 ***	0.0000	−0.0104	−0.0071
	(0.0081)	(0.0081)	(0.0249)	(0.0194)	(0.0176)
First-stage					
tech_p	−8.223 ***	−8.3849 ***	−10.9730 ***	−7.6270 ***	−10.8952 ***
	(1.0988)	(1.3708)	(1.8974)	(1.4940)	(1.2600)
rob	0.0041 ***	0.0100	0.0027 *	0.0066 **	0.0011
	(0.0015)	(0.0074)	(0.0014)	(0.0027)	(0.0016)
Controls	Yes	Yes	Yes	Yes	Yes
Industry FE	Yes	Yes	Yes	Yes	Yes
County FE	Yes	Yes	Yes	Yes	Yes
Year FE	Yes	Yes	Yes	Yes	Yes
F statistic	28.55	35.89	17.23	13.09	37.53
Hanse J *p* value	0.227	0.290	0.313	0.281	0.797
Observations	15755	3828	4419	2969	4078

Notes: Robust standard errors, reported in parentheses, are clustered by industry and county. ***, **, and * denote significance at the 1%, 5%, and 10% levels, respectively. The detailed version, which includes the estimated results of the control variables, is presented in Table A6.

**Table 6:** Results of propensity score matching.

	Variables	Treated	Controls	Diff	S.E.	T Value
Unmatched	underedu	2.0454	1.8426	0.2028	0.0248	8.19
	overedu	1.7112	1.8317	−0.1205	0.0188	−6.41
K-nearest neighbor matching (1:1)	underedu	1.9269	2.1582	−0.2313	0.0860	−2.69 ***
	overedu	1.7131	1.7320	−0.0189	0.0306	−0.62
K-nearest neighbor matching (1:4)	underedu	1.9269	2.1095	−0.1826	0.0606	−3.01 ***
	overedu	1.7131	1.7203	−0.0072	0.0255	−0.28
Radius matching	underedu	1.9269	2.1917	−0.2649	0.0505	−5.25 ***
	overedu	1.7131	1.7287	−0.0156	0.0226	−0.69
Local linear regression matching	underedu	1.9267	2.1358	−0.2088	0.0860	−2.43 ***
	overedu	1.7131	1.7317	−0.0186	0.0306	−0.61
Kernel matching	underedu	1.9269	2.1851	−0.2583	0.0506	−5.11 ***
	overedu	1.7131	1.7291	−0.0160	0.0226	−0.71

Notes: The above table lists the estimates of the female sample aged 18–35. *** denote significance at the 1% levels.

**Table 7:** Results of mechanism analysis.

Variables	*lnWage*	*lnSaving*	*Job_sat*	*Wellbing*	*Prom*	*Prom_adm*	*Prom_tech*
	(1)	(2)	(3)	(4)	(5)	(6)	(7)
$d_{underedu}$	0.0285 *	0.0331	−0.0011	−0.0125	0.0222 ***	0.0137	0.0138 **
	(0.0148)	(0.0697)	(0.0117)	(0.0192)	(0.0061)	(0.0080)	(0.0062)
edu	0.0518 ***	0.0628	−0.0042	0.0168	0.0396 ***	0.0182 ***	0.0255 ***
	(0.0068)	(0.0710)	(0.0049)	(0.0204)	(0.0055)	(0.0048)	(0.0068)
Controls	Yes	Yes	Yes	Yes	Yes	Yes	Yes
Industry FE	Yes	Yes	Yes	Yes	Yes	Yes	Yes
County FE	Yes	Yes	Yes	Yes	Yes	Yes	Yes
Year FE	Yes	Yes	Yes	Yes	Yes	Yes	Yes
Observations	2595	2421	3699	3699	2373	2373	2373
R-squared	0.3458	0.2360	0.1597	0.1595	0.2290	0.1757	0.1951

Notes: The above table lists the estimates of the female sample aged 18–35. Robust standard errors, reported in parentheses, are clustered by industry and county. ***, **, and * denote significance at the 1%, 5%, and 10% levels, respectively. The detailed version, which includes the estimated results of the control variables, is presented in Table A7.

**Table 8:** Results of mechanism analysis.

Variables	*Low_edu*	*High_edu*	*Low_income*	*Middle_income*	*High_income*
	(1)	(2)	(3)	(4)	(5)
$d_{underedu}$	−0.0201 ***	0.0001	−0.0295 ***	−0.0073	0.0009
	(0.0052)	(0.0263)	(0.0080)	(0.0079)	(0.0073)
edu	−0.0294 ***	0.0061	−0.0134	−0.0154 *	−0.0050
	(0.0080)	(0.0081)	(0.0080)	(0.0078)	(0.0059)
Controls	Yes	Yes	Yes	Yes	Yes
Industry FE	Yes	Yes	Yes	Yes	Yes
County FE	Yes	Yes	Yes	Yes	Yes
Year FE	Yes	Yes	Yes	Yes	Yes
Observations	1762	1865	835	1083	1644
R-squared	0.2677	0.2208	0.3349	0.3331	0.2489
**Variables**	***Self-employed***	***Employed***	***Public_sector***	***Private_sector***	
	**(6)**	**(7)**	**(8)**	**(9)**	
$d_{underedu}$	0.0213 *	−0.0107 **	0.0177	−0.0134 ***	
	(0.0105)	(0.0041)	(0.0220)	(0.0045)	
edu	0.0159 *	−0.0087	0.0089	−0.0111 **	
	(0.0087)	(0.0054)	(0.0117)	(0.0047)	
Controls	Yes	Yes	Yes	Yes	
Industry FE	Yes	Yes	Yes	Yes	
County FE	Yes	Yes	Yes	Yes	
Year FE	Yes	Yes	Yes	Yes	
Observations	525	3107	655	2382	
R-squared	0.3759	0.2087	0.3265	0.2378	

Notes: The above table lists the estimates of the female sample aged 18–35. Robust standard errors, reported in parentheses, are clustered by industry and county. ***, **, and * denote significance at the 1%, 5%, and 10% levels, respectively. The detailed version, which includes the estimated results of the control variables, is presented in Table A8.

## Data Availability

Data used in this research can be downloaded from the CFPS homepage (URL: http://www.isss.pku.edu.cn/cfps/, accessed on 17 May 2021).

## Appendix A

**Table A1:** *redu* estimates within each group.

	Eastern Region		Central Region		Western Region	
	<1980	≥1980	<1980	≥1980	<1980	≥1980
Heads of government agencies and public institutions	11.73	14.43	12.16	14.70	11.54	14.40
CEO	9.23	11.91	9.21	11.33	7.77	11.06
Technical staff	12.28	15.06	12.76	14.50	11.61	14.10
Medical staff	12.39	14.44	11.94	14.46	12.36	13.61
Economic and commercial staff	13.16	14.38	12.72	14.38	12.60	13.87
Financial staff	12.11	14.50	12.56	13.93	11.89	13.52
Legal professional	15.33	16.00	15.25	15.83	14.71	15.75
Teaching staff	13.99	14.35	13.75	13.87	14.24	14.69
Arts and sports professionals	11.23	13.94	10.91	13.84	8.80	13.41
Administrative staff	11.73	14.11	12.84	13.83	12.41	13.96
Security and fire service personnel	8.24	11.20	9.64	11.66	8.35	11.84
Post and telecommunications worker	10.65	12.01	11.32	12.38	7.50	10.91
Other office worker	9.33	12.70	9.49	11.71	9.86	10.67
Salesperson	9.03	11.30	9.00	10.90	7.85	10.50
Warehouse personnel	9.40	10.99	9.61	10.58	8.71	10.36
Catering service staff	7.24	9.29	7.45	8.98	6.47	8.34
Tourism and entertainment service personnel	8.57	10.23	7.97	10.40	7.92	9.85
Transportation service personnel	10.12	12.33	8.40	12.52	8.44	11.97
Social service worker	7.33	10.64	7.79	10.34	6.69	9.32
Planting industry production personnel	5.35	8.08	5.07	8.02	4.03	6.13
Forestry production and wildlife protection personnel	5.07	7.34	6.29	9.57	7.22	6.00
Animal husbandry production personnel	6.13	7.61	6.11	8.48	4.73	8.75
Fishery production personnel	5.07	7.15	6.53	8.00	7.95	12.75
Geological exploration and mineral extraction personnel	8.80	12.00	8.35	10.33	6.63	10.21
Metal smelting personnel	8.72	10.96	9.95	9.75	9.47	10.25
Chemical product manufacturing personnel	8.39	12.53	9.48	11.88	6.00	10.87
Machinery and equipment manufacturing personnel	7.73	10.29	8.76	9.27	8.52	8.91
Mechanical and electrical product assembly worker	7.68	9.98	7.91	10.20	8.83	9.46
Mechanical equipment repair personnel	9.26	10.71	9.01	10.68	7.89	9.14
Electrical equipment installation, operation, maintenance and power supply personnel	9.33	11.28	10.28	9.55	9.04	9.98
Electronic component and equipment manufacturing, assembly, troubleshooting and maintenance worker	6.56	9.43	7.65	9.91	9.30	9.24
Rubber and plastic product manufacturing worker	6.29	9.07	7.35	9.48	6.20	8.90
Textile, knitting, printing and dyeing worker	6.07	8.02	6.58	8.38	8.37	8.60
Cutting, sewing and leather and fur product processing and manufacturing worker	6.88	7.69	6.79	8.38	6.18	7.67
Food handler	8.05	9.74	8.08	10.80	6.13	9.68
Wood processing worker	7.52	8.36	7.63	8.86	5.73	8.13
Cement and cement products preparation and processing worker	6.81	9.12	7.54	9.16	6.88	8.00
Glass fitter	6.75	9.36	7.20	8.00	4.94	9.30
Film and television production worker	7.75	11.00	7.50	14.00	4.50	8.40
Printer	8.89	10.82	11.25	9.90	12.00	9.30
Craft and arts production worker	7.06	9.12	5.33	8.29	6.96	7.68
Engineering construction worker	7.02	8.79	6.71	8.59	6.28	8.10
Transport equipment operator and related worker	8.55	9.43	8.54	9.44	7.58	8.62
Inspection personnel	10.00	11.83	10.03	11.23	10.63	11.23
Other production and transport equipment operator and related worker	6.66	8.75	6.78	8.27	5.16	7.62

**Table A2:** Correlation matrix.

	1	2	3	4	5	6	7	8	9	10	11	12
intended children	1											
$d_{underedu}$	0.08	1										
$d_{overedu}$	0.04	−0.14	1									
*edu*	−0.12	−0.57	0.44	1								
gender	0.04	−0.03	0.09	−0.04	1							
age	0.07	0.03	0.02	−0.23	0.04	1						
hukou	0.09	0.16	−0.22	−0.41	0.03	−0.19	1					
residence	−0.09	−0.13	0.15	0.29	−0.07	0.11	−0.42	1				
health status	0.00	−0.03	−0.01	0.05	0.08	−0.24	0.08	−0.07	1			
household income	−0.05	−0.13	0.13	0.32	−0.04	0.03	−0.24	0.22	−0.02	1		
employment break	−0.02	−0.07	0.04	0.09	0.03	0.21	−0.07	0.07	−0.09	0.17	1	
family size	0.16	0.06	−0.09	−0.14	−0.01	−0.06	0.14	−0.23	0.03	0.06	−0.02	1
proportion of old people	−0.01	−0.02	0.00	−0.04	0.01	0.12	−0.03	−0.01	−0.02	0.00	0.01	0.20
marital status	0.06	0.04	−0.02	−0.17	−0.06	0.53	−0.08	0.06	−0.14	0.05	0.14	0.12
IQV index	−0.03	−0.03	0.12	0.14	−0.04	−0.01	−0.12	0.10	0.01	0.08	0.04	−0.06
views on gender norms	0.12	0.07	−0.06	−0.15	0.13	0.04	0.17	−0.12	0.06	−0.02	0.14	0.11
fertility status	0.12	0.06	−0.05	−0.23	−0.03	0.59	−0.05	0.03	−0.15	0.05	0.17	0.22
migration	−0.01	0.00	0.02	0.06	−0.01	−0.08	0.02	0.08	0.02	0.08	−0.05	−0.13
wage	−0.03	−0.12	0.14	0.27	0.20	0.08	−0.16	0.13	0.01	0.38	0.20	−0.07
household savings	−0.06	−0.08	0.08	0.19	−0.01	0.02	−0.10	0.15	−0.04	0.28	0.14	−0.10
job satisfaction	−0.01	−0.02	−0.01	0.10	−0.03	0.00	−0.03	0.01	0.13	0.10	0.07	−0.01
subjective well-being	−0.02	−0.05	−0.01	0.08	−0.05	−0.07	−0.03	0.02	0.21	0.10	−0.03	0.06
willing to promotion	−0.01	−0.08	0.08	0.30	0.05	−0.23	−0.09	0.04	0.06	0.10	0.02	−0.03
employment type	−0.09	−0.03	0.06	0.16	−0.03	−0.10	−0.10	0.02	0.01	−0.01	−0.02	−0.07
	**13**	**14**	**15**	**16**	**17**	**18**	**19**	**20**	**21**	**22**	**23**	**24**
proportion of old people	1											
marital status	0.02	1										
IQV index	0.02	−0.03	1									
views on gender norms	0.00	0.02	−0.04	1								
fertility status	0.00	0.72	−0.06	0.05	1							
migration	−0.06	−0.01	0.04	−0.01	−0.05	1						
wage	−0.02	0.06	0.03	0.06	0.05	0.09	1					
household savings	−0.01	−0.01	0.04	0.03	0.00	0.04	0.17	1				
job satisfaction	−0.01	0.01	0.05	0.09	0.00	0.00	0.11	0.05	1			
subjective well-being	−0.02	0.00	0.01	0.04	0.02	0.00	0.02	0.03	0.20	1		
willing to promotion	−0.03	−0.15	0.05	−0.01	−0.17	0.02	0.14	0.09	0.09	0.06	1	
employment type	0.01	−0.10	−0.08	−0.04	−0.12	0.02	0.10	0.08	−0.04	−0.01	0.13	1

**Table A3:** Benchmark results (detailed version).

Variables		18–35		36–50	
	All	Female	Male	Female	Male
	(1)	(2)	(3)	(4)	(5)
**Panel A**					
$d_{underedu}$	0.0008	−0.0092 **	0.0088	0.0089	−0.0082
	(0.0032)	(0.0040)	(0.0088)	(0.0060)	(0.0105)
edu	−0.0043	−0.0090 **	−0.0102	−0.0011	−0.0010
	(0.0027)	(0.0041)	(0.0069)	(0.0049)	(0.0067)
gender	0.0434 ***				
	(0.0099)				
hukou	0.0368	0.0016	−0.0161	0.1207 ***	0.0407 *
	(0.0230)	(0.0209)	(0.0749)	(0.0290)	(0.0217)
age	0.0062 ***	0.0131 ***	0.0062 ***	0.0085 ***	0.0029
	(0.0008)	(0.0015)	(0.0021)	(0.0021)	(0.0019)
residence	−0.0181	−0.0023	−0.0303	0.0042	−0.0276
	(0.0128)	(0.0176)	(0.0240)	(0.0245)	(0.0232)
health status	0.0050	0.0242 ***	0.0069	0.0040	−0.0098
	(0.0048)	(0.0075)	(0.0091)	(0.0126)	(0.0066)
household income	0.0054	0.0116	0.0105	0.0043	−0.0142
	(0.0066)	(0.0096)	(0.0129)	(0.0113)	(0.0125)
employment break	0.0078	0.0053	0.0098	0.0132	−0.0139
	(0.0145)	(0.0185)	(0.0200)	(0.0190)	(0.0289)
family size	0.0280 ***	0.0233 ***	0.0156 *	0.0415 ***	0.0475 ***
	(0.0040)	(0.0064)	(0.0088)	(0.0107)	(0.0078)
proportion of old people	−0.1330 ***	−0.1742 **	−0.0699	−0.3242 ***	−0.0051
	(0.0453)	(0.0658)	(0.1612)	(0.0784)	(0.0794)
marital status—married	0.1049 ***	0.0357	0.1205 ***	0.3100	0.3875 ***
	(0.0173)	(0.0255)	(0.0141)	(0.1846)	(0.0903)
marital status—divorced	−0.0229	−0.0778	−0.0212	0.2339	0.2186 *
	(0.0344)	(0.0661)	(0.0482)	(0.1781)	(0.1121)
IQV index	0.0211	−0.4135	0.2792	−0.0043	−0.0093
	(0.1582)	(0.3727)	(0.3122)	(0.2608)	(0.2114)
Industry FE	Yes	Yes	Yes	Yes	Yes
County FE	Yes	Yes	Yes	Yes	Yes
Year FE	Yes	Yes	Yes	Yes	Yes
Observations	14,535	3699	3852	2939	3626
R-squared	0.1997	0.2141	0.2133	0.2944	0.2839
**Panel B**					
$d_{overedu}$	0.0030	0.0007	0.0052	−0.0015	0.0043
	(0.0027)	(0.0045)	(0.0052)	(0.0087)	(0.0051)
edu	−0.0020	−0.0114 ***	−0.0028	0.0019	0.0019
	(0.0023)	(0.0027)	(0.0043)	(0.0047)	(0.0047)
gender	0.0473 ***				
	(0.0091)				
hukou	0.0418 **	0.0067	0.0152	0.1190 ***	0.0384
	(0.0158)	(0.0213)	(0.0309)	(0.0264)	(0.0227)
age	0.0059 ***	0.0128 ***	0.0061 ***	0.0093 ***	0.0005
	(0.0008)	(0.0014)	(0.0021)	(0.0019)	(0.0022)
residence	−0.0138	0.0152	−0.0176	−0.0225	−0.0385
	(0.0123)	(0.0182)	(0.0216)	(0.0302)	(0.0228)
health status	0.0050	0.0183 **	0.0098	0.0005	−0.0038
	(0.0041)	(0.0067)	(0.0097)	(0.0140)	(0.0067)
household income	0.0079	0.0151 *	−0.0015	0.0206	−0.0085
	(0.0066)	(0.0086)	(0.0164)	(0.0154)	(0.0129)
employment break	0.0109	0.0318	−0.0103	0.0235	−0.0178
	(0.0146)	(0.0223)	(0.0241)	(0.0174)	(0.0258)
family size	0.0276 ***	0.0201 **	0.0181 **	0.0370 ***	0.0452 ***
	(0.0031)	(0.0081)	(0.0074)	(0.0116)	(0.0080)
proportion of old people	−0.1029 **	−0.1053	−0.0652	−0.2742 ***	−0.0049
	(0.0442)	(0.0710)	(0.1568)	(0.0797)	(0.0640)
marital status—married	0.1039 ***	0.0148	0.1436 ***	0.3275 *	0.2640 **
	(0.0157)	(0.0308)	(0.0210)	(0.1668)	(0.0991)
marital status—divorced	−0.0597	−0.1025	−0.0265	0.2085	0.0827
	(0.0393)	(0.0686)	(0.0418)	(0.1740)	(0.1416)
IQV index	−0.0884	−0.5004 *	−0.0189	−0.2514	0.0771
	(0.1551)	(0.2740)	(0.2920)	(0.2747)	(0.2099)
Industry FE	Yes	Yes	Yes	Yes	Yes
County FE	Yes	Yes	Yes	Yes	Yes
Year FE	Yes	Yes	Yes	Yes	Yes
Observations	15,830	3854	4437	2983	4095
R-squared	0.1879	0.2060	0.1912	0.2761	0.2675

Notes: Robust standard errors, reported in parentheses, are clustered by industry and county. ***, **, and * denote significance at the 1%, 5%, and 10% levels, respectively.

**Table A4:** Results of replacing the explanatory variable (detailed version).

Variables	Dummy Variables		Mode Method	
	Female	Male	Female	Male
	(1)	(2)	(3)	(4)
**Panel A**				
underedu	−0.0318 *	0.0220		
	(0.0182)	(0.0446)		
$d_{underedu}$			−0.0152 **	−0.0018
			(0.0054)	(0.0099)
edu	−0.0079 *	−0.0124 *	−0.0154 ***	−0.0194 **
	(0.0043)	(0.0061)	(0.0037)	(0.0090)
hukou	0.0029	−0.0189	−0.0128	−0.0602
	(0.0212)	(0.0734)	(0.0253)	(0.1006)
age	0.0132 ***	0.0061 ***	0.0141 ***	0.0047 *
	(0.0015)	(0.0021)	(0.0024)	(0.0023)
residence	−0.0021	−0.0301	−0.0007	−0.0443
	(0.0176)	(0.0240)	(0.0214)	(0.0265)
health status	0.0246 ***	0.0069	0.0250 **	0.0001
	(0.0076)	(0.0092)	(0.0094)	(0.0108)
household income	0.0112	0.0108	0.0211	−0.0027
	(0.0094)	(0.0129)	(0.0123)	(0.0143)
employment break	0.0053	0.0094	−0.0066	−0.0019
	(0.0185)	(0.0202)	(0.0189)	(0.0205)
family size	0.0236 ***	0.0155 *	0.0186 **	0.0202 *
	(0.0065)	(0.0088)	(0.0076)	(0.0106)
proportion of old people	−0.1758 **	−0.0692	−0.2017 ***	−0.0503
	(0.0652)	(0.1608)	(0.0670)	(0.1637)
marital status—married	0.0363	0.1200 ***	0.0519 *	0.1557 ***
	(0.0254)	(0.0133)	(0.0295)	(0.0204)
marital status—divorced	−0.0769	−0.0216	−0.0525	0.0318
	(0.0664)	(0.0486)	(0.0900)	(0.0873)
IQV index	−0.4534	0.3284	0.0158	0.5215
	(0.3664)	(0.3218)	(0.3836)	(0.4818)
Industry FE	Yes	Yes	Yes	Yes
County FE	Yes	Yes	Yes	Yes
Year FE	Yes	Yes	Yes	Yes
Observations	3699	3852	2833	2824
R-squared	0.2137	0.2131	0.2308	0.2367
**Panel B**				
overedu	0.0017	0.0209		
	(0.0186)	(0.0211)		
$d_{overedu}$			0.0026	0.0053
			(0.0035)	(0.0036)
edu	−0.0113 ***	−0.0022	−0.0105 ***	−0.0043
	(0.0030)	(0.0041)	(0.0034)	(0.0044)
hukou	0.0067	0.0155	0.0037	0.0043
	(0.0213)	(0.0309)	(0.0267)	(0.0338)
age	0.0128 ***	0.0063 ***	0.0111 ***	0.0067 ***
	(0.0014)	(0.0021)	(0.0016)	(0.0022)
residence	0.0152	−0.0176	0.0225	−0.0175
	(0.0182)	(0.0216)	(0.0174)	(0.0217)
health status	0.0183 **	0.0100	0.0280 ***	0.0126
	(0.0067)	(0.0097)	(0.0055)	(0.0103)
household income	0.0151 *	−0.0017	0.0129	0.0108
	(0.0085)	(0.0164)	(0.0098)	(0.0185)
employment break	0.0318	−0.0102	0.0213	−0.0043
	(0.0223)	(0.0241)	(0.0209)	(0.0225)
family size	0.0201 **	0.0181 **	0.0182 *	0.0205 **
	(0.0081)	(0.0074)	(0.0089)	(0.0079)
proportion of old people	−0.1053	−0.0652	−0.1112 *	−0.1023
	(0.0712)	(0.1566)	(0.0629)	(0.1696)
marital status—married	0.0148	0.1433 ***	0.0030	0.1551 ***
	(0.0309)	(0.0208)	(0.0365)	(0.0231)
marital status—divorced	−0.1025	−0.0267	−0.0695	0.0065
	(0.0686)	(0.0414)	(0.0589)	(0.0640)
IQV index	−0.4979 *	−0.0178	−0.7465 **	−0.2074
	(0.2717)	(0.2900)	(0.3212)	(0.3646)
Industry FE	Yes	Yes	Yes	Yes
County FE	Yes	Yes	Yes	Yes
Year FE	Yes	Yes	Yes	Yes
Observations	3854	4437	3260	3881
R-squared	0.2060	0.1911	0.2144	0.1953

Notes: The above table lists the estimates of the sample aged 18–35. Robust standard errors, reported in parentheses, are clustered by industry and county. ***, **, and * denote significance at the 1%, 5%, and 10% levels, respectively.

**Table A5:** Discussion about the omitted variable bias (detailed version).

Variables	Female				Male			
	(1)	(2)	(3)	(4)	(5)	(6)	(7)	(8)
**Panel A**								
$d_{underedu}$	−0.0087 *	−0.0090 **	−0.0088 **	−0.0081 *	0.0094	0.0089	0.0115	0.0122
	(0.0043)	(0.0039)	(0.0038)	(0.0039)	(0.0088)	(0.0089)	(0.0104)	(0.0104)
edu	−0.0063	−0.0081 *	−0.0092 **	−0.0063	−0.0074	−0.0097	−0.0113	−0.0083
	(0.0040)	(0.0042)	(0.0039)	(0.0040)	(0.0067)	(0.0070)	(0.0085)	(0.0086)
gen_view	0.0370 ***			0.0281 ***	0.0512 ***			0.0463 ***
	(0.0061)			(0.0065)	(0.0083)			(0.0098)
fer_status		0.0732 **		0.0744 **		0.0585		0.0474
		(0.0300)		(0.0268)		(0.0338)		(0.0292)
mig_status			0.0242	0.0259			−0.0244	−0.0222
			(0.0371)	(0.0364)			(0.0466)	(0.0446)
hukou	0.0028	0.0000	−0.0062	−0.0063	−0.0250	−0.0184	−0.0434	−0.0530
	(0.0210)	(0.0211)	(0.0243)	(0.0246)	(0.0740)	(0.0745)	(0.0788)	(0.0775)
age	0.0131 ***	0.0115 ***	0.0117 ***	0.0101 ***	0.0069 ***	0.0051 **	0.0071 **	0.0069 **
	(0.0015)	(0.0018)	(0.0021)	(0.0025)	(0.0022)	(0.0022)	(0.0026)	(0.0029)
residence	−0.0044	−0.0033	−0.0060	−0.0079	−0.0295	−0.0311	−0.0381	−0.0376
	(0.0171)	(0.0183)	(0.0147)	(0.0147)	(0.0247)	(0.0239)	(0.0237)	(0.0242)
health status	0.0195 **	0.0244 ***	0.0193 ***	0.0167 **	0.0007	0.0069	0.0124	0.0067
	(0.0079)	(0.0076)	(0.0065)	(0.0074)	(0.0085)	(0.0091)	(0.0107)	(0.0099)
household income	0.0113	0.0122	0.0102	0.0099	0.0119	0.0113	0.0238	0.0264
	(0.0089)	(0.0102)	(0.0162)	(0.0167)	(0.0120)	(0.0130)	(0.0159)	(0.0154)
employment break	0.0053	0.0038	−0.0197	−0.0215	0.0060	0.0083	0.0120	0.0062
	(0.0178)	(0.0193)	(0.0216)	(0.0224)	(0.0189)	(0.0198)	(0.0188)	(0.0189)
family size	0.0230 ***	0.0209 ***	0.0200 ***	0.0174 ***	0.0149	0.0135	0.0112	0.0089
	(0.0059)	(0.0059)	(0.0062)	(0.0055)	(0.0091)	(0.0089)	(0.0083)	(0.0086)
proportion of old people	−0.1634 **	−0.1651 **	−0.1486 ***	−0.1319 **	−0.0612	−0.0551	−0.0382	−0.0144
	(0.0662)	(0.0687)	(0.0483)	(0.0496)	(0.1632)	(0.1613)	(0.1536)	(0.1547)
marital status—married	0.0281	−0.0120	0.0324	−0.0223	0.1162 ***	0.0810 **	0.1072 ***	0.0742 **
	(0.0258)	(0.0380)	(0.0282)	(0.0371)	(0.0137)	(0.0305)	(0.0174)	(0.0298)
marital status—divorced	−0.0679	−0.1282 *	−0.0679	−0.1151	−0.0072	−0.0578	0.0054	−0.0103
	(0.0655)	(0.0628)	(0.0765)	(0.0734)	(0.0541)	(0.0410)	(0.0532)	(0.0566)
IQV index	−0.3963	−0.4398	−0.5374	−0.5617	0.2864	0.2867	0.0124	0.0358
	(0.3425)	(0.3590)	(0.3848)	(0.3492)	(0.3061)	(0.3129)	(0.2260)	(0.2207)
Industry FE	Yes	Yes	Yes	Yes	Yes	Yes	Yes	Yes
County FE	Yes	Yes	Yes	Yes	Yes	Yes	Yes	Yes
Year FE	Yes	Yes	Yes	Yes	Yes	Yes	Yes	Yes
Observations	3698	3699	3320	3319	3852	3852	3331	3331
R-squared	0.2207	0.2155	0.2172	0.2226	0.2207	0.2138	0.2298	0.2366
**Panel B**								
$d_{overedu}$	0.0008	0.0010	−0.0010	−0.0004	0.0048	0.0052	0.0073	0.0066
	(0.0045)	(0.0045)	(0.0050)	(0.0049)	(0.0053)	(0.0051)	(0.0055)	(0.0056)
edu	−0.0081 ***	−0.0106 ***	−0.0119 ***	−0.0081 ***	−0.0004	−0.0023	−0.0049	−0.0017
	(0.0024)	(0.0027)	(0.0028)	(0.0027)	(0.0044)	(0.0043)	(0.0057)	(0.0057)
gen_view	0.0451 ***			0.0369 ***	0.0495 ***			0.0454 ***
	(0.0076)			(0.0083)	(0.0090)			(0.0101)
fer_status		0.0544 *		0.0560 *		0.0539 *		0.0622 *
		(0.0308)		(0.0298)		(0.0289)		(0.0323)
mig_status			0.0238	0.0201			−0.0154	−0.0165
			(0.0329)	(0.0326)			(0.0399)	(0.0387)
hukou	0.0086	0.0058	−0.0024	−0.0011	0.0050	0.0139	−0.0136	−0.0239
	(0.0221)	(0.0214)	(0.0228)	(0.0234)	(0.0292)	(0.0308)	(0.0323)	(0.0306)
age	0.0127 ***	0.0115 ***	0.0111 ***	0.0097 ***	0.0068 ***	0.0050 **	0.0070 ***	0.0063 **
	(0.0014)	(0.0017)	(0.0017)	(0.0021)	(0.0022)	(0.0023)	(0.0024)	(0.0027)
residence	0.0123	0.0142	0.0064	0.0039	−0.0182	−0.0182	−0.0191	−0.0200
	(0.0174)	(0.0181)	(0.0139)	(0.0136)	(0.0217)	(0.0214)	(0.0215)	(0.0213)
health status	0.0117 *	0.0186 **	0.0140 *	0.0092	0.0038	0.0098	0.0138	0.0081
	(0.0067)	(0.0066)	(0.0075)	(0.0067)	(0.0094)	(0.0097)	(0.0121)	(0.0117)
household income	0.0160 *	0.0155 *	0.0166	0.0171	−0.0011	−0.0009	0.0151	0.0169
	(0.0080)	(0.0088)	(0.0138)	(0.0140)	(0.0162)	(0.0165)	(0.0135)	(0.0133)
employment break	0.0322	0.0305	0.0172	0.0155	−0.0141	−0.0120	−0.0115	−0.0181
	(0.0222)	(0.0229)	(0.0276)	(0.0285)	(0.0230)	(0.0241)	(0.0230)	(0.0224)
family size	0.0192 **	0.0180 **	0.0172 **	0.0145 **	0.0167 **	0.0159 **	0.0143 *	0.0101
	(0.0074)	(0.0077)	(0.0071)	(0.0064)	(0.0077)	(0.0070)	(0.0073)	(0.0070)
proportion of old people	−0.0895	−0.0990	−0.1092 **	−0.0902 *	−0.0541	−0.0513	−0.0147	0.0148
	(0.0702)	(0.0732)	(0.0493)	(0.0505)	(0.1591)	(0.1540)	(0.1659)	(0.1650)
marital status—married	0.0047	−0.0184	0.0119	−0.0304	0.1387 ***	0.1091 ***	0.1385 ***	0.0962 ***
	(0.0320)	(0.0404)	(0.0317)	(0.0417)	(0.0194)	(0.0289)	(0.0232)	(0.0291)
marital status—divorced	−0.0918	−0.1365 **	−0.0903	−0.1211	−0.0174	−0.0574	−0.0162	−0.0402
	(0.0673)	(0.0624)	(0.0834)	(0.0787)	(0.0468)	(0.0407)	(0.0553)	(0.0603)
IQV index	−0.4450 *	−0.5135 *	−0.6547 **	−0.6283 **	0.0069	−0.0155	−0.3011	−0.2560
	(0.2565)	(0.2705)	(0.2610)	(0.2482)	(0.2884)	(0.2919)	(0.3047)	(0.3054)
Industry FE	Yes	Yes	Yes	Yes	Yes	Yes	Yes	Yes
County FE	Yes	Yes	Yes	Yes	Yes	Yes	Yes	Yes
Year FE	Yes	Yes	Yes	Yes	Yes	Yes	Yes	Yes
Observations	3854	3854	3451	3451	4437	4437	3825	3825
R-squared	0.2152	0.2069	0.2099	0.2171	0.1983	0.1916	0.1988	0.2059

Notes: The above table lists the estimates of the sample aged 18–35. Robust standard errors, reported in parentheses, are clustered by industry and county. ***, **, and * denote significance at the 1%, 5%, and 10% levels, respectively.

**Table A6:** Results of 2SLS (detailed version).

Variables		18–35		36–50	
	All	Female	Male	Female	Male
	(1)	(2)	(3)	(4)	(5)
**Panel A**					
$d_{underedu}$	0.0375	−0.0651 ***	0.0376	0.0026	−0.0227
	(0.0246)	(0.0146)	(0.0771)	(0.0596)	(0.0476)
edu	0.0121	−0.0336 ***	0.0022	−0.0043	−0.0082
	(0.0116)	(0.0084)	(0.0312)	(0.0290)	(0.0249)
gender	0.0469 ***				
	(0.0092)				
hukou	0.0531 *	−0.0181	0.0000	0.1161 **	0.0348
	(0.0276)	(0.0233)	(0.0703)	(0.0440)	(0.0330)
age	0.0080 ***	0.0117 ***	0.0068 **	0.0082 **	0.0022
	(0.0015)	(0.0022)	(0.0024)	(0.0037)	(0.0025)
residence	−0.0204	−0.0074	−0.0318	0.0050	−0.0252
	(0.0126)	(0.0160)	(0.0237)	(0.0250)	(0.0223)
health status	0.0063	0.0233 ***	0.0071	0.0049	0.0099
	(0.0046)	(0.0073)	(0.0094)	(0.0129)	(0.0068)
household income	0.0013	0.0159	0.0084	0.0062	−0.0117
	(0.0071)	(0.0099)	(0.0155)	(0.0171)	(0.0147)
employment break	0.0081	0.0024	0.0125	0.0135	−0.0136
	(0.0143)	(0.0181)	(0.0191)	(0.0172)	(0.0295)
family size	0.0284 ***	0.0205 ***	0.0156	0.0412 ***	0.0466 ***
	(0.0039)	(0.0062)	(0.0095)	(0.0106)	(0.0076)
proportion of old people	−0.1245 **	−0.1767 **	−0.0723	−0.3229 ***	−0.0015
	(0.0450)	(0.0706)	(0.1622)	(0.0927)	(0.0845)
marital status—married	0.1072 ***	0.0330	0.1213 ***	0.3103	0.3942 ***
	(0.0177)	(0.0247)	(0.0137)	(0.1854)	(0.0887)
marital status—divorced	−0.0208	−0.0748	−0.0229	0.2363	0.2300 *
	(0.0339)	(0.0634)	(0.0449)	(0.1925)	(0.1147)
IQV index	−0.1152	−0.1664	0.0902	0.0773	0.1101
	(0.1981)	(0.5417)	(0.7741)	(0.2467)	(0.2892)
First-stage					
tech_p	7.4118 ***	8.2680 ***	7.5607 ***	8.6787 ***	7.6972 ***
	(0.9073)	(1.4122)	(1.5857)	(1.2210)	(0.9190)
rob	−0.0025	−0.0058	−0.0012	−0.0079 **	−0.0010
	0.0015	(0.0049)	(0.0020)	(0.0040)	(0.0010)
Industry FE	Yes	Yes	Yes	Yes	Yes
County FE	Yes	Yes	Yes	Yes	Yes
Year FE	Yes	Yes	Yes	Yes	Yes
F statistic	34.06	17.68	14.18	26.98	46.38
Hanse J *p* value	0.252	0.190	0.506	0.181	0.493
Observations	14,488	3685	3841	2927	3615
**Panel B**					
$d_{overedu}$	−0.0130	0.0331	−0.0010	0.0342	0.0212
	(0.0172)	(0.0196)	(0.0509)	(0.0583)	(0.0299)
edu	0.0048	−0.0250 ***	0.0000	−0.0104	−0.0071
	(0.0081)	(0.0081)	(0.0249)	(0.0194)	(0.0176)
gender	0.0521 ***				
	(0.0102)				
hukou	0.0419 **	0.0045	0.0167	0.1113 ***	0.0384
	(0.0154)	(0.0218)	(0.0292)	(0.0279)	(0.0222)
age	0.0066 ***	0.0118 ***	0.0064 **	0.0088 ***	−0.0000
	(0.0011)	(0.0016)	(0.0029)	(0.0020)	(0.0023)
residence	−0.0161	0.0155	−0.0184	−0.0221	−0.0382
	(0.0118)	(0.0192)	(0.0213)	(0.0283)	(0.0233)
health status	0.0051	0.0190 ***	0.0099	0.0011	0.0038
	(0.0042)	(0.0061)	(0.0105)	(0.0138)	(0.0069)
household income	0.0072	0.0170 *	−0.0029	0.0224	−0.0075
	(0.0068)	(0.0084)	(0.0176)	(0.0165)	(0.0112)
employment break	0.0105	0.0331	−0.0096	0.0196	−0.0172
	(0.0142)	(0.0203)	(0.0233)	(0.0167)	(0.0266)
family size	0.0271 ***	0.0196 **	0.0177 **	0.0380 ***	0.0446 ***
	(0.0030)	(0.0079)	(0.0076)	(0.0117)	(0.0074)
proportion of old people	−0.1024 **	−0.1035	−0.0705	−0.2855 ***	−0.0052
	(0.0428)	(0.0706)	(0.1567)	(0.0896)	(0.0668)
marital status—married	0.1060 ***	0.0166	0.1426 ***	0.3292 *	0.2726 **
	(0.0156)	(0.0294)	(0.0218)	(0.1613)	(0.0963)
marital status—divorced	−0.0576	−0.0906	−0.0286	0.2094	0.0954
	(0.0384)	(0.0705)	(0.0401)	(0.1689)	(0.1430)
IQV index	−0.0307	−0.6008	0.0224	−0.3553	0.1558
	(0.1207)	(0.4523)	(0.3176)	(0.4729)	(0.2262)
First-stage					
tech_p	−8.223 ***	−8.3849 ***	−10.9730 ***	−7.6270 ***	−10.8952 ***
	(1.0988)	(1.3708)	(1.8974)	(1.4940)	(1.2600)
rob	0.0041 ***	0.0100	0.0027 *	0.0066 **	0.0011
	(0.0015)	(0.0074)	(0.0014)	(0.0027)	(0.0016)
Industry FE	Yes	Yes	Yes	Yes	Yes
County FE	Yes	Yes	Yes	Yes	Yes
Year FE	Yes	Yes	Yes	Yes	Yes
F statistic	28.55	35.89	17.23	13.09	37.53
Hanse J *p* value	0.227	0.290	0.313	0.281	0.797
Observations	15,755	3828	4419	2969	4078

Notes: Robust standard errors, reported in parentheses, are clustered by industry and county. ***, **, and * denote significance at the 1%, 5%, and 10% levels, respectively.

**Table A7:** Results of mechanism analysis (detailed version).

Variables	*lnWage*	*lnSaving*	*Job_sat*	*Wellbing*	*Prom*	*Prom_adm*	*Prom_tech*
	(1)	(2)	(3)	(4)	(5)	(6)	(7)
$d_{underedu}$	0.0285 *	0.0331	−0.0011	−0.0125	0.0222 ***	0.0137	0.0138 **
	(0.0148)	(0.0697)	(0.0117)	(0.0192)	(0.0061)	(0.0080)	(0.0062)
edu	0.0518 ***	0.0628	−0.0042	0.0168	0.0396 ***	0.0182 ***	0.0255 ***
	(0.0068)	(0.0710)	(0.0049)	(0.0204)	(0.0055)	(0.0048)	(0.0068)
hukou	−0.0438	0.1631	−0.0760 **	−0.1078	−0.0429	−0.0687 **	−0.0274
	(0.0491)	(0.3268)	(0.0352)	(0.0859)	(0.0330)	(0.0319)	(0.0472)
age	0.0348 ***	0.0275	−0.0104 *	−0.0151 *	−0.0022	−0.0054 **	0.0009
	(0.0107)	(0.0254)	(0.0053)	(0.0080)	(0.0026)	(0.0021)	(0.0028)
residence	−0.0299	0.7235 *	−0.0433 **	−0.0410	−0.0289	−0.0183	−0.0293
	(0.0540)	(0.3544)	(0.0205)	(0.0754)	(0.0279)	(0.0231)	(0.0293)
health status	0.0648 ***	0.1477	0.1057 ***	0.4002 ***	0.0040	0.0084	0.0106
	(0.0146)	(0.0900)	(0.0141)	(0.0480)	(0.0079)	(0.0107)	(0.0091)
household income	0.2005 ***	1.3507 ***	0.0655 ***	0.1341 **	0.0211	0.0060	0.0170
	(0.0450)	(0.1896)	(0.0183)	(0.0497)	(0.0122)	(0.0141)	(0.0150)
employment break	0.1267 **	0.3399	−0.0496	−0.0606	−0.0178	0.0103	−0.0126
	(0.0493)	(0.2791)	(0.0313)	(0.0517)	(0.0297)	(0.0252)	(0.0329)
family size	−0.0069	−0.2782 ***	−0.0153	0.0173	−0.0036	−0.0036	−0.0058
	(0.0167)	(0.0764)	(0.0117)	(0.0252)	(0.0050)	(0.0064)	(0.0047)
proportion of old people	−0.0207	0.7383	−0.0646	−0.2353	0.0892	0.0656	0.0061
	(0.0832)	(1.1938)	(0.1298)	(0.2097)	(0.0603)	(0.0749)	(0.0700)
marital status—married	−0.1585 **	0.1278	0.1045 *	0.0860	−0.0522	−0.0341	−0.0792 *
	(0.0558)	(0.2175)	(0.0599)	(0.0983)	(0.0376)	(0.0291)	(0.0389)
marital status—divorced	−0.0570	−1.5227 **	0.3988 ***	−1.1026 **	0.1209	0.1343	0.1841 *
	(0.1133)	(0.6526)	(0.0865)	(0.3927)	(0.1005)	(0.1045)	(0.0984)
IQV index	1.2213	−2.4240	0.0582	−0.2848	0.5929	1.5164 ***	−0.4233
	(0.7678)	(5.3424)	(0.4385)	(0.9470)	(0.5002)	(0.4517)	(0.7328)
Industry FE	Yes	Yes	Yes	Yes	Yes	Yes	Yes
County FE	Yes	Yes	Yes	Yes	Yes	Yes	Yes
Year FE	Yes	Yes	Yes	Yes	Yes	Yes	Yes
Observations	2595	2421	3699	3699	2373	2373	2373
R-squared	0.3458	0.2360	0.1597	0.1595	0.2290	0.1757	0.1951

Notes: The above table lists the estimates of the female sample aged 18–35. Robust standard errors, reported in parentheses, are clustered by industry and county. ***, **, and * denote significance at the 1%, 5%, and 10% levels, respectively.

**Table A8:** Results of mechanism analysis (detailed version).

Variables	*Low_edu*	*High_edu*	*Low_income*	*Middle_income*	*High_income*
	(1)	(2)	(3)	(4)	(5)
$d_{underedu}$	−0.0201 ***	0.0001	−0.0295 ***	−0.0073	0.0009
	(0.0052)	(0.0263)	(0.0080)	(0.0079)	(0.0073)
edu	−0.0294 ***	0.0061	−0.0134	−0.0154 *	−0.0050
	(0.0080)	(0.0081)	(0.0080)	(0.0078)	(0.0059)
hukou	−0.0024	0.0110	−0.0993 *	0.0598	0.0014
	(0.0528)	(0.0318)	(0.0567)	(0.0572)	(0.0273)
age	0.0108 ***	0.0129 ***	0.0137 **	0.0113 ***	0.0147 ***
	(0.0024)	(0.0034)	(0.0058)	(0.0037)	(0.0033)
residence	0.0306 *	−0.0212	−0.0332	0.0157	−0.0080
	(0.0158)	(0.0260)	(0.0407)	(0.0251)	(0.0231)
health status	0.0173	0.0239 *	0.0303	0.0089	0.0330 **
	(0.0113)	(0.0130)	(0.0209)	(0.0103)	(0.0121)
household income	0.0196	−0.0105	−0.0455	0.1730 **	0.0405
	(0.0150)	(0.0158)	(0.0284)	(0.0764)	(0.0516)
employment break	−0.0263	0.0344	0.0055	0.0268	−0.0012
	(0.0304)	(0.0275)	(0.0347)	(0.0320)	(0.0310)
family size	0.0170 **	0.0343 ***	0.0133	0.0450 ***	0.0231 **
	(0.0079)	(0.0094)	(0.0108)	(0.0131)	(0.0107)
proportion of old people	−0.1786 **	−0.1399	−0.3551 **	−0.2017	−0.1007
	(0.0645)	(0.1138)	(0.1608)	(0.1677)	(0.1546)
marital status—married	0.0475	0.0328	0.0813	0.0019	0.0401
	(0.0366)	(0.0345)	(0.0731)	(0.0437)	(0.0364)
marital status—divorced	−0.0341	−0.1483	−0.0309	−0.0015	−0.0889
	(0.0904)	(0.1179)	(0.1184)	(0.1507)	(0.1429)
IQV index	0.2397	−1.3294 *	−0.4861	−0.1254	−0.3043
	(0.4046)	(0.6888)	(0.6451)	(0.5827)	(0.4496)
Industry FE	Yes	Yes	Yes	Yes	Yes
County FE	Yes	Yes	Yes	Yes	Yes
Year FE	Yes	Yes	Yes	Yes	Yes
Observations	1762	1865	835	1083	1644
R-squared	0.2677	0.2208	0.3349	0.3331	0.2489
**Variables**	***Self-employed***	***Employed***	***Public_sector***	***Private_sector***	
	**(6)**	**(7)**	**(8)**	**(9)**	
$d_{underedu}$	0.0213 *	−0.0107 **	0.0177	−0.0134 ***	
	(0.0105)	(0.0041)	(0.0220)	(0.0045)	
edu	0.0159 *	−0.0087	0.0089	−0.0111 **	
	(0.0087)	(0.0054)	(0.0117)	(0.0047)	
hukou	0.1123	−0.0073	0.0431	−0.0167	
	(0.0991)	(0.0236)	(0.0590)	(0.0275)	
age	0.0201 ***	0.0128 ***	0.0173 **	0.0123 ***	
	(0.0061)	(0.0018)	(0.0079)	(0.0022)	
residence	0.0547	−0.0114	−0.0082	−0.0182	
	(0.0562)	(0.0182)	(0.0486)	(0.0193)	
health status	0.0483	0.0190 *	0.0791 ***	0.0038	
	(0.0316)	(0.0092)	(0.0269)	(0.0108)	
household income	0.0414	0.0073	0.0541	0.0045	
	(0.0331)	(0.0105)	(0.0424)	(0.0099)	
employment break	−0.0130	0.0092	−0.0626	0.0169	
	(0.0377)	(0.0201)	(0.0436)	(0.0220)	
family size	0.0262 **	0.0220 **	0.0258 *	0.0231 **	
	(0.0117)	(0.0085)	(0.0140)	(0.0095)	
proportion of old people	−0.4119 *	−0.1304 *	−0.0808	−0.0957	
	(0.1911)	(0.0724)	(0.1953)	(0.0769)	
marital status—married	−0.0437	0.0446	0.0767	0.0282	
	(0.0868)	(0.0276)	(0.0570)	(0.0298)	
marital status—divorced	−0.2458	−0.0621	−0.1046	−0.0595	
	(0.3250)	(0.0625)	(0.2596)	(0.0564)	
IQV index	−2.6165 **	−0.1432	−0.5567	−0.2544	
	(0.9618)	(0.4225)	(0.8738)	(0.5581)	
Industry FE	Yes	Yes	Yes	Yes	
County FE	Yes	Yes	Yes	Yes	
Year FE	Yes	Yes	Yes	Yes	
Observations	525	3107	655	2382	
R-squared	0.3759	0.2087	0.3265	0.2378	

Notes: The above table lists the estimates of the female sample aged 18–35. Robust standard errors, reported in parentheses, are clustered by industry and county. ***, **, and * denote significance at the 1%, 5%, and 10% levels, respectively.
